# Implementation of a quality by design approach into cell line and process development for recombinant protein production

**DOI:** 10.1186/1753-6561-9-S9-P79

**Published:** 2015-12-14

**Authors:** Caroline Noack, Anne Schüler, Markus Wieland

**Affiliations:** 1 Cellca GmbH, Laupheim, D-88471, Germany

## Background

Cellca is a recognized leader in cell line and upstream process development for large-scale protein production of biopharmaceuticals (e.g. antibodies) in mammalian cells and offers a unique platform technology which is characterized by high efficiency regarding productivity, scalability, and protein quality. As the market for therapeutic proteins is steadily growing, the need to optimize methods for the generation of mammalian cell lines is steadily increasing. In this context, important aspects are to express recombinant proteins not only at high levels and in an efficient cost-effective manner, but also with the desired protein quality attributes such as the glycoprofile. Cellca's unique platform technology enables the expression of antibody products with different protein quality attributes using only a single host cell line. We identified three steps to select, optimize and confirm scalability of a high-producing cell line expressing proteins with the desired quality profile. Thus, with this study we aim to contribute to a better understanding of how quality in terms of expressing proteins with pre-defined glycoprofiles can be built into a cell line and process development process.

## Materials and methods

A recombinant CHO DG44 cell line expressing an IgG1 antibody was developed using Cellca´s proprietary platform technology. For this purpose, different cell clones were generated and subsequently evaluated in a platform fed-batch process at shake flask scale for their producer cell line potential. Product quality analysis was implemented into the development process early on and allowed selection of the clone with the most desirable product quality profile. For media and process variation studies, cells were cultured in fed-batch mode in both shake flask and bioreactor scale using Cellca's platform process and proprietary cell culture media. Cell densities and culture viabilities were obtained using a CASY cell counter. Antibody concentrations were determined by Protein A HPLC. Bioreactor experiments in 200 L and 1000 L scale were performed at Rentschler Biotechnologie GmbH (Laupheim, Germany). Analytical data of product quality was provided by Protagen Protein Services GmbH (Dortmund, Germany). The Galactosylation index was calculated according to Kunkel et al [1].

## Results

**Figure 1:**
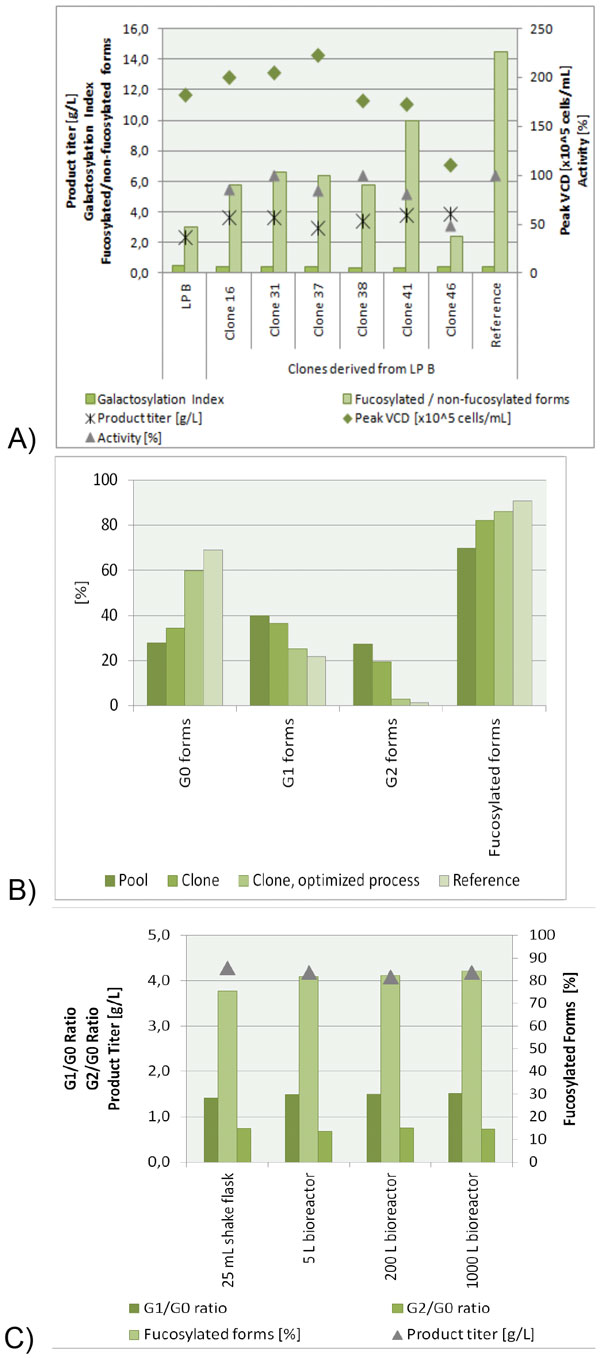
**A) Six clones derived from cell pool LPB were analyzed for fed-batch performance and protein quality attributes of the model IgG1 antibody**. B) Effective clone selection as well as media and process optimization enables to match the N-glycosylation pattern of a reference protein. C) Scalability of the fed-batch process from shake flask to bioreactor scale has been demonstrated for different clones and is exemplarily shown for an IgG1 antibody.

The basis for all development work herein described was the Cellca CHO DG44 host cell line which offers a broad flexibility expressing different antibody products regarding several quality attributes. We identified three steps to select, optimize and confirm the scalability of a high-producing cell line expressing proteins with the desired quality profile. In the first step, suitable cell clones needed to be selected: In a quality by design approach it is crucial to have a sufficient amount of high producing cell clones available. Based on the fed-batch performance in a standard process, 48 high producing clones were analyzed for the desired target protein quality profile (see exemplary results in Figure 1A). The selection of the clones with the most promising quality profile was facilitated by the knowledge of media and process optimization capabilities. The protein quality profile of an antibody can be actively influenced by fed-batch process and culture media design. Thus, in the second step, several conditions, like media components, process parameters or feeding regimen, were tested and conditions to increase or reduce galactosylation profiles could be identified. They have been successfully applied for different antibody products at Cellca. Selection of the optimal process and media conditions can therefore help to obtain the desired protein quality (Figure 1B). In the third step, the scalability of the fed-batch process optimized in 25 mL shake flask was studied. As shake flask processes at Cellca are designed to serve as bioreactor scale-down model, the scale-up to different bioreactors up to 1000 L volume could be easily performed without further optimization or extensive adjustments. During scale-up, not only productivity and cell growth were comparable to the shake flask model, but also protein quality attributes of the produced antibody (Figure 1C). This proven scalability is a key factor for optimization in small scales.

## Conclusions

It becomes increasingly important to express recombinant proteins not only at high levels and in an efficient cost-effective manner, but also with the desired protein quality attributes, e.g. the glycoprofile. In this study, we presented an approach of how quality in terms of expressing proteins with pre-defined glycoprofiles can be built into a cell line and process development process.

A main building block and starting point for cell line development is Cellca's CHO DG44 host cell line, which can be flexibly used to produce antibodies that cover a broad range of various quality attributes. Thus, there is no need to screen several host cell lines for expression of the correct target antibody characteristics, as one cell line and the technology platform associated with it is sufficient to express different antibody products each with their individual protein quality attributes. In the present study, we developed a recombinant cell line expressing a monoclonal human IgG1 antibody. Implementing a quality by design approach for pool and clone selection allowed to pre-select cell pools based on their glycan profile before single cell cloning. Subsequently, high-producing cell clones that expressed the target antibody with a desirable protein quality profile were identified in a standard fed-batch process and used for further studies. We showed that the galactosylation profile can be influenced, both towards higher and lower galactosylation levels, by media and process variation. As depicted in Figure 1B, effective clone selection as well as media and process optimization enabled to match the N-glycosylation pattern of the expressed IgG1 antibody to a reference protein. The optimized production process was scalable from 25 mL shake flask up to 1000 L bioreactor levels regarding growth, productivity and glycosylation. Implementing the quality by design approach into cell line and process development can thus enable to successfully target a desired protein quality profile.

## Acknowledgements

This project was supported by the German Federal Ministry for Economics and Technology (BMWi) through a ZIM (Central innovation program for medium-sized companies) project grant. We would like to thank Martin Blüggel of Protagen Protein Services GmbH, Dortmund, for the performance of product quality analytics. Furthermore, the author would like to thank Rentschler Biotechnologie GmbH, Laupheim, in particular Sven Reiser for the performance of the bioreactor studies in 200 L and 1000 L scale.
